# An ensemble deep learning approach to evaluate haptic delay from a single trial EEG data

**DOI:** 10.3389/frobt.2022.1013043

**Published:** 2022-09-27

**Authors:** Haneen Alsuradi, Mohamad Eid

**Affiliations:** ^1^ Engineering Division, New York University Abu Dhabi, Abu Dhabi, United Arab Emirates; ^2^ Tandon School of Engineering, New York University, New York, NY, United States

**Keywords:** neurohaptics, haptics, deep learning, wavelet transform, CNN, convolutional neural network, EEG

## Abstract

Haptic technologies are becoming increasingly valuable in Human-Computer interaction systems as they provide means of physical interaction with a remote or virtual environment. One of the persistent challenges in tele-haptic systems, communicating haptic information over a computer network, is the synchrony of the delivered haptic information with the rest of the sensory modalities. Delayed haptic feedback can have serious implications on the user performance and overall experience. Limited research efforts have been devoted to studying the implication of haptic delay on the human neural response and relating it to the overall haptic experience. Deep learning could offer autonomous brain activity interpretation in response to a haptic experience such as haptic delay. In this work, we propose an ensemble of 2D CNN and transformer models that is capable of detecting the presence and redseverity of haptic delay from a single-trial Electroencephalography data. Two EEG-based experiments involving visuo-haptic interaction tasks are proposed. The first experiment aims to collect data for detecting the presence of haptic delay during discrete force feedback using a bouncing ball on a racket simulation, while the second aims to collect data for detecting the severity level (none, mild, moderate, severe) of the haptic delay during continuous force feedback via grasping/releasing of an object in a bucket. The ensemble model showed a promising performance with an accuracy of 0.9142 ± 0.0157 for detecting haptic delay during discrete force feedback and 0.6625 ± 0.0067 for classifying the severity of haptic delay during continuous force feedback (4 levels). These results were obtained based on training the model with raw EEG data as well as their wavelet transform using several wavelet kernels. This study is a step forward towards developing cognitive evaluation of the user experience while interaction with haptic interfaces.

## 1 Introduction

Haptic technologies are becoming increasingly valuable in human-computer interaction as they allow humans to interact with a virtual or remote environments using a simulated sense of touch. For instance, telehaptic systems are assisted with haptic feedback allowing users to perform delicate and complex tasks in remote or unreachable environment while concurrently perceiving the physical aspects of the remote environment (Xu, 2017). One of the persistent challenges in telehaptic systems is the synchrony of the delivered information across modalities (visual, auditory, and haptics). Strong demands are placed on the communication network due to the transmission of haptic information and thus, haptic delay is probable (Van Den Berg et al., 2017). Through psychophysical investigations, the effects of haptic delay on the user experience and/or performance have garnered substantial attention. There is ample evidence that haptic delay interferes with task completion time (Ferrell, 1965), impairs performance (Tatematsu et al., 2011) and deceitfully manipulates the haptic sensation (Knorlein et al., 2009). Depending on the application and the kind of haptic interaction, detection thresholds for haptic delays can range widely, from 20 to 200 ms (Vogels, 2004). In other words, whether the user is experiencing a discrete force, continuous force, or vibrotactile feedback and whether they are involved in an active or passive interaction can all affect how haptic delay is experienced.

Our comprehension of the human perception of haptic delay is therefore necessary for the development of reliable and resilient haptic devices designed for usage across computer networks. Conventional methods for evaluating the experience of haptic delay are based on self-reporting and/or behavioural analysis (psychophysical studies). However, these methods are subject to biases due to previous user experience and/or experimental context, prone to social pressure, and hard to reproduce. An emerging field called neurohaptics uses brain imaging techniques to examine the intricate neural representations triggered by haptic stimulation (Alsuradi et al., 2020a). Electroencephalography (EEG) is one of the mostly used tools for this purpose mainly due to their compatibility with other electronic devices in the vicinity of the EEG system. Compared to other neuroimaging methods such as the functional magnetic resonance imaging (fMRI), EEG has a high temporal resolution which is crucial for studying a time related perceptual quality such as the haptic delay.

EEG data are rich in information over multiple dimensions, namely time and space (i.e., across electrodes scattered on the scalp). Consequently, EEG data can be used to train deep learning models which could possibly allow for autonomous brain activity interpretation in response to physical interaction, leading to the quantification of the perceived haptic experience (Miura et al., 2014; Alsuradi et al., 2020b). EEG motor imagery signals have been extensively used to train deep learning models to distinguish between imagined right and left limb movements (Tabar and Halici, 2016; Al-Saegh et al., 2021). Another popular example is using deep learning to identify the emotional state of users from their EEG data (Chen et al., 2019; Donmez and Ozkurt, 2019; Babushkin et al., 2021). On the contrary, limited number of studies have employed deep learning in neurohaptics. An attempt was made to classify the surface texture during active exploration task (Eldeeb et al., 2020) on a single EEG trial basis using Support Vector Machine (SVM) with features that are manually extracted from the raw EEG data. Another study developed a CNN model to identify the type of haptic interaction (passive vs. active) during visuo-haptic task on a single EEG trial basis as well (Alsuradi and Eid, 2021). Most of these studies target haptic experiences related to the physical properties of the stimulus or the movement. However, only few studies were found to explore the usage of deep learning in analyzing high order cognitive functions associated with haptic experience and none tackled haptic delay. Evaluating the cognitive experience of haptic delay during a haptic interaction over a computer network from a single trial EEG data remains unanswered.

In our previous studies, we explored and identified the prominent neural signatures associated with detecting haptic delay (Alsuradi et al., 2021) as well as estimating the level of the haptic delay (Alsuradi et al., 2022). The first study aimed to understand the neural correlates that encodes the presence of haptic delay during a discrete force feedback under passive and active haptic interactions. P200 feature in the central cortex, commonly tied to sensory attention, was found to be modulated under the presence of haptic delay regardless of the interaction type (passive vs. active). Midfrontal theta power was also found to encode the perception of haptic delay. The second study on the other hand aimed to identify the neural correlates associated with the level of haptic delay. Interestingly, midfrontal theta power was found to significantly differ between the different levels of delay, suggesting an encoding mechanism. Midfrontal theta power is generally associated with neural processes related to conflict processing and resolution (Cohen and Donner, 2013; Arrighi et al., 2016); haptic delay can be thought as a form of sensory conflict. In both studies, sensory correlates, such as the post movement beta rebound (PMBR) (Kilavik et al., 2013), were found to be delayed with an amount proportional to the introduced delay level. All these studies indicate that EEG data contain prominent information about the haptic delay.

The main objective of this work is to use single-trial EEG data collected during the two previously mentioned studies for the purpose of building reliable deep learning models that are able to detect the presence and the severity levels of haptic delay. The contributions of this manuscript are listed below:

• Developing a model that is aimed to detect the presence of haptic delay during a discrete force feedback stimulation while being resilient to the type of haptic interaction (passive vs. active).
• Developing another model that is aimed to differentiate between the different levels of haptic delay (no delay, mild delay, moderate delay and severe delay) during a continuous force feedback stimulation.
• Both models are developed with the characteristics of: 1) operating on a single EEG trial 2) avoiding the use of any crafted features.

Towards this end, we explore using an ensemble of Deep ConvNet (Schirrmeister et al., 2017) and the state of the art model, transformer (Vaswani et al., 2017), in utilizing EEG data and several of its representations using wavelet transform.

## 2 Materials and methods

We designed two experiments to examine the possibility of detecting the presence and amount of haptic delay from EEG data, respectively. The first experiment involves two types of interaction (passive and active) where the haptic delay is perceived during a *discrete* haptic feedback. In the second experiment, four levels of delay were introduced (no delay, mild delay, moderate delay, major delay) during a *continuous* haptic feedback stimulation.

### 2.1 Experiment 1: Detecting the presence of delay during discrete haptic feedback

#### 2.1.1 Participants

In experiment 1, nineteen participants have been asked to take part in the study (10 females and nine males), where 90% of them are undergraduate students aged between 18 and 25 years. All subjects were right-handed and used their right hand to complete the experiment. In addition, participants had normal or corrected-to-normal eyesight. The study was conducted out in compliance with the Declaration of Helsinki, following its norms and regulations, and with an authorized protocol by the New York University Abu Dhabi Institutional Review Board (IRB: #HRPP-2019-120). Before joining in this study, all subjects provided written informed consent in compliance with IRB standards. Participants received around 30 USD compensation voucher for their participation in the study.

#### 2.1.2 Task

Participants were told that they would be taking part in a haptic-visual activity in which they would bounce a tennis ball with a racket controlled by a haptic device. Participants were requested to sit on a chair in front of a computer display and use their right hand to grip the stylus of a haptic device (Geomagic Touch, 3D Systems, United States). The Unity game engine version 2018.4.5f1 (Unity technologies, United States) and Openhaptics Unity toolkit were used to create the game (3D Systems, United States). Subjects had to complete passive and active tasks while experiencing synchronous or asynchronous visuo-haptic stimulation. Participants lifted the racket up to bounce the ball, which was initially motionless above the racket, during the active task. During the passive task, however, the racket was held passively, and a thumb button push on the haptic device caused the tennis ball to come loose and collide with the racket. When the ball collided with the racket, force feedback was felt. The haptic collision might be delivered concurrently with the visual collision (synchronous) or 220 ms after the visual collision (asynchronous).

**FIGURE 1:**
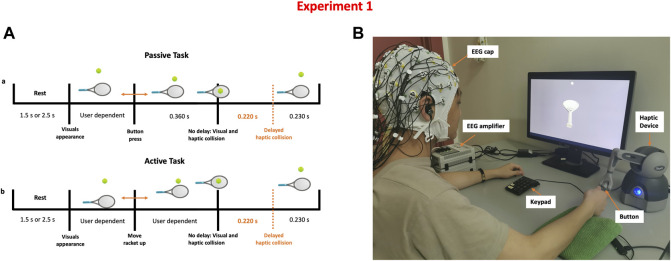
Detecting the presence of haptic delay experiment **(A)** Task time-line of a single trial under the passive and active haptic interactions **(B)** Experimental setup showing a participant correctly holding the stylus to control the racket shown on the screen. Synchronous and delayed haptic feedback are delivered through the stylus.

The timeline of a single trial is shown in Figure 1. The trial starts with a blank screen presentation for 1.5 or 2.5 s (randomized), followed by a single bouncing motion. Each participant completed 200 trials in total, evenly distributed across four experimental conditions: Passive No Delay (PND), Passive Delay (PD), Active No Delay (AND), Active Delay (AD). Trials were distributed over ten runs, each with 20 trials. The first five runs were done in the passive mode, while the latter five were done in the active mode. During a single run, 10 trials had synchronous visuo-haptic stimulation while the other 10 trials involved haptic delay. The sequence of trials within a single run was randomized.

### 2.2 Experiment 2: Classification of the level of haptic delay during a continuous haptic feedback

#### 2.2.1 Participants

A total of thirty-four subjects have been invited to participate in experiment 2 (17 females and 17 males), where the majority of them (97%) are undergraduate students aged between 18 and 25 years. Participants were all right-handed and completed the experiment using their right hand. Participants also had normal or corrected-to-normal vision. The protocol used in this study was approved by the Institutional Review Board at New York University Abu Dhabi (IRB: #HRPP-2021-17) and followed the Declaration of Helsinki’s rules and regulations. Before participating in this study, all individuals completed an informed consent form in compliance with the IRB’s principles. Each participant received an Amazon voucher worth 30 dollars (USD) for their active participation. This experiment was done post COVID-19 spread, and thus, to protect participants from COVID-19, many precautionary and preventative steps were followed, including keeping physical distance, using surgical masks and gloves before any contact with the participants, sanitizing the haptic device after each usage, and completing a symptom check form to verify participants were not sick.

#### 2.2.2 Task

Participants were instructed to perform a simulated pick and release task using a computer screen and a haptic device. The task required participants to use the haptic device (Geomagic Touch, 3D systems, United States) to pick up a cylindrical-shaped object displayed on the screen and move it towards a bucket where it should be released. Once the object is picked, participants experienced a force feedback that simulates the weight of the object. Thus, both visual and haptic feedback were provided; the visual feedback coming from the computer screen and the haptic feedback coming from the haptic device. Once the object is released, the force feedback is stopped and the weight of the object is no longer felt. However, depending on the condition, the force feedback could be activated for an additional amount of time. This interval of time is referred to as a haptic delay. There are four degrees of haptic delay: *D*_0_ = 0 ms (No delay), *D*_1_ = 120 ms (Mild delay), *D*_2_ = 250 ms (Moderate delay), and *D*_3_ = 400 ms (Severe delay). After performing a pilot study, these delay thresholds were determined (Alsuradi et al., 2022).

**FIGURE 2:**
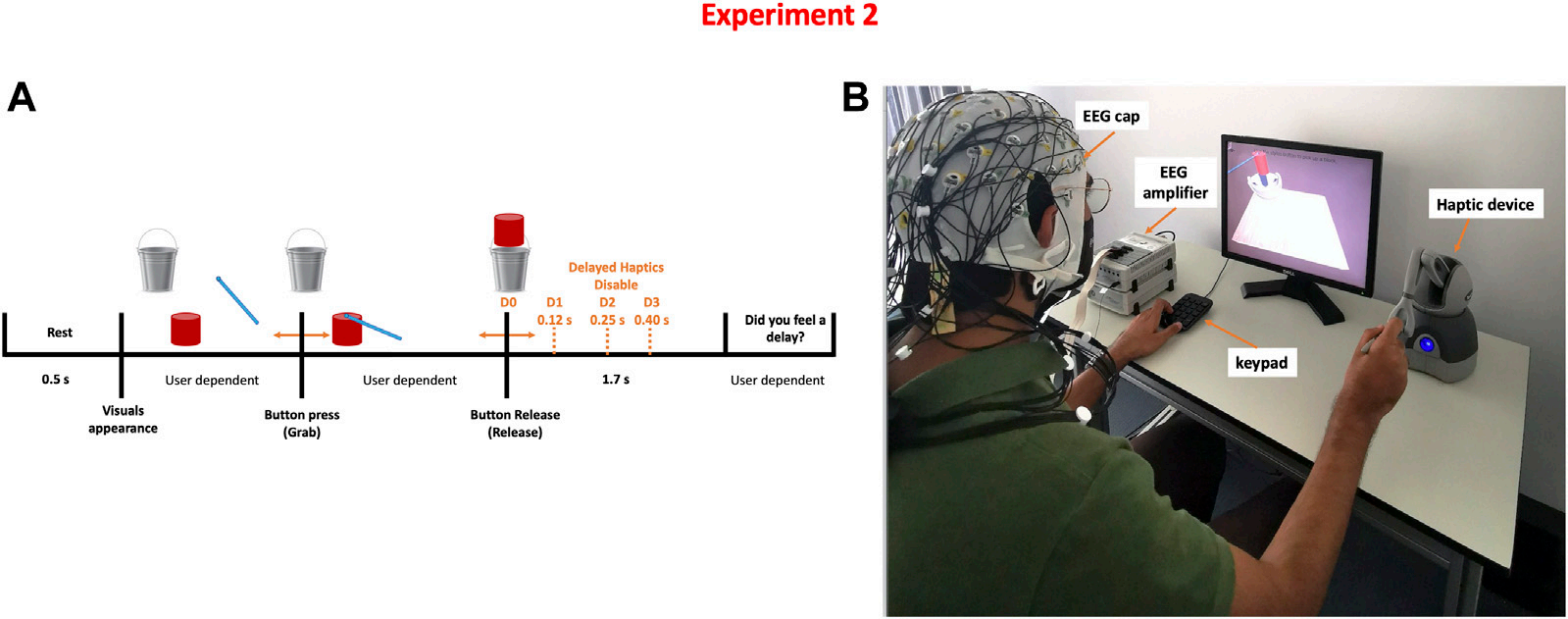
Detecting the level of haptic delay experiment **(A)** Task time-line of a single trial where four possible delay levels can be perceived **(B)** Experimental setup showing a participant correctly holding the stylus to pick up and carry the object.

The task timeline is shown in Figure 2. The trial starts with a 0.5 s rest time followed by a single grab and release action; the duration of a single trial is thus variable and user dependent. Ten runs were conducted per participant; each run consisted of 16 grab and release trials (four distinct levels of haptic delay × four repeats) organized in a counterbalanced fashion by a Latin square order (Grant, 1948). Each participant completed 160 trials in total, evenly split between the four delay conditions. Similar to experiment 1, Unity game engine version 2018.4.5f1 (Unity technologies, United States) and Openhaptics Unity toolkit were used to create the task (3D Systems, United States).

### 2.3 EEG data

#### 2.3.1 Acquisition and pre-processing

During the experiment, EEG data were captured at a sampling rate of 1 kHz using a BrainAmps amplifier (BrainAmps Standard, Brain Products, Germany). To handle the acquisition process and monitor the electrode connection quality, the Brain Vision Recorder software (BVR; Version 1.21.0201 Brain Products, Germany) was utilized. We employed 64 Ag/AgCL active electrodes with noise suppression and amplification built-in to the readout electronics. The EEG cap was placed on participants’ heads using the 10-20 international positioning method, with the Cz electrode at the vertex of the head. The online reference was positioned at FCz, whereas the ground electrode was positioned at FPz. We kept the connection impedance between the electrode and the scalp below 1,510 kΩ to guarantee high-quality signal recordings.

MATLAB version 2021a (MathWorks, United States) and EEGLAB toolbox (v14.1.2) (Delorme and Makeig, 2004) were used to pre-process and analyze the data offline. The data was filtered between 0.1 and 50 Hz using a sinc FIR filter with a Hamming window. To minimize power line noise centered around 50 Hz, a notch filter was utilized. The EEG data were subjected to the Artifact Subspace Reconstruction (ASR) (Kothe and Jung, 2016) technique to eliminate high-amplitude artifacts such muscle activity, eye blinks, and movements, as well as to detect and reject heavily contaminated trials. The data were re-referenced using the Common Average Referencing (CAR) technique (Lakshmi et al., 2014), and the data from the online reference FCz were retained. Any residual ocular and muscle artifacts were isolated and removed using Independent Component Analysis (ICA).

#### 2.3.2 Epoching and baseline correction

• Experiment 1: For each trial, the time between the emergence of visuals and the visual collision (Δ_1_) was computed. Trials with Δ_1_ more than 4 standard deviations away from the mean were removed in order to preserve a consistent behavioral trend across trials. The data were epoched between -200 and 1,000 ms around the visual collision. Time-domain epoched trials were baseline corrected using a baseline between -200 and the onset, which is the visual collision event.
• Experiment 2: For each trial, the time between pick up and release (Δ_2_) was computed to estimate its duration. Short trials demonstrate that the subject grabbed up and released the object quickly, most likely by accident. The pick and release exercise should take at least 2–3 s for a well-trained individual. As a result, extremely short trials with *Δ*_2_ ≤ 1 s were eliminated. The rest of the trials were then epoched from -200 to 1,000 ms around the object release event. Time-domain epoched trials were baseline-corrected using a baseline between -200 and the onset, which is the visual object release event.

### 2.4 Feature extraction

Raw EEG data carries plenty of information towards encoding the presence and amount of haptic delay that can be extracted directly by state of the art deep learning models. However, embedding extracted prominent features in addition to the raw EEG data can drastically enhance the accuracy of classification. It has been shown that wavelet transform is highly effective in extracting features from raw EEG data because it deals greatly with the non-stationary behavior of EEG signals (Amin et al., 2015). Wavelet coefficients have been reported as useful features for normal EEG analysis as well as in clinical applications (Yazdani et al., 2009; Garry et al., 2013). Wavelet coefficients offer simultaneous localization of neural activation in time and frequency domains which is indeed required to extract haptic delay information. Haptic delay is encoded in several frequency bands like theta, alpha and beta bands (Alsuradi et al., 2021, 2022).

**FIGURE 3:**
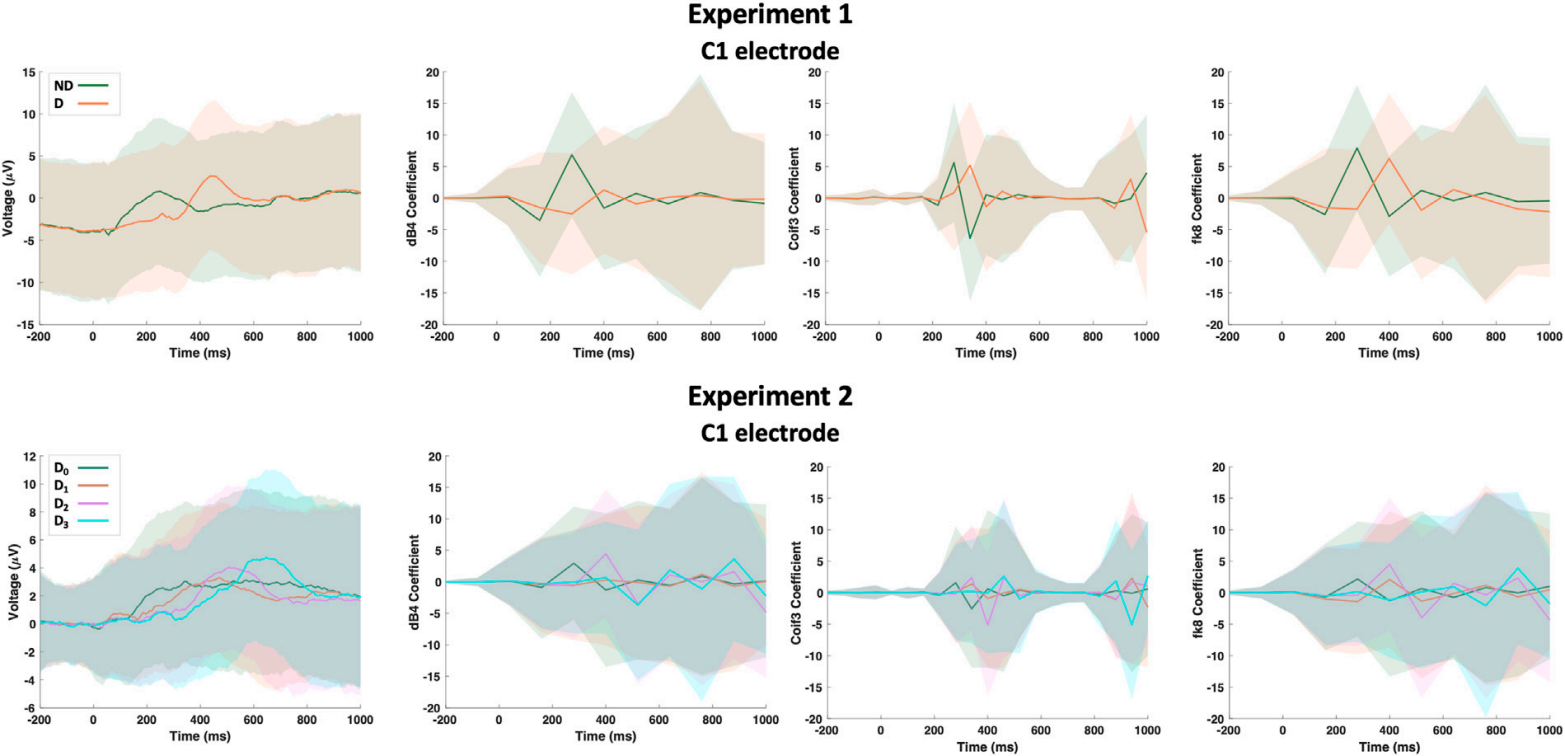
A sample of the dataset showing the raw EEG data and its wavelet transform based on Daubechies-4 (dB4), Coiflet-3 (Coif3) and Fejér-Korovkin-8 (fk8) wavelets for C1 electrode. Hard lines show the mean of the trials while shaded regions shows the standard deviation.

Raw EEG data from both experiments were first down-sampled from 1kHz to 125 Hz such that a single epoch is of length *N*_1_. Next, the following wavelet functions were used for the wavelet transform analysis and feature extraction: Daubechies-4 (dB4), Daubechies-20 (dB20), Coiflet-1 (Coif1), Coiflet-3 (Coif3), Symlet-10 (Sym10), Fejér-Korovkin-8 (fk8), Biorthogonal-6.8 (bior6.8) and Reverse biorthogonal-6.8 (rbio6.8). These wavelet transforms were selected empirically based on their impact on the overall detection accuracy. Each epoch per electrode went under all the previously mentioned wavelet transforms and all the transforms were concatenated to form a single 1-D array of length *N*_2_. Figure 3 shows a sample of the dataset including raw and transformed for C1 electrode. Two input data matrices were formed one for each experiment; an input data matrix, *X*, can be formed with dimensions of trials (Tr), sequence length (*N*_1_ + *N*_2_), and electrodes (*E*). Based on the model’s choice, the input data can be reshaped accordingly. Data from experiment 1 are classified as either No Delay (*ND*) or delayed (*D*) regardless of the activity type (passive vs. active). On the other hand, data from experiment 2 are classified as either No Delay (*D*_0_), mildly delayed (*D*_1_), moderately delayed (*D*_2_), or severely delayed (*D*_3_). To facilitate referring to the above parameters in the remainder of the manuscript, we list them and a few other important parameters in Table 1.

**TABLE 1:** Dataset related parameters.

Parameter	Symbol	Experiment 1	Experiment 2
Subjects	*Sub*	19	34
Electrodes	*E*	60	60
Classes	*C*	2	4
Total trials	Tr	3,753	5,418
Raw data sequence length	*N*_1_	150	150
Wavelet transform sequence length	*N*_2_	171	171

### 2.5 Dataset

The final dataset that was used for training and validating the proposed deep learning models consists of the raw EEG data concatenated with the wavelet transformed versions of the raw EEG data. The transformer model expects time-series data which is one-dimensional in nature, while the 2D CNN expects two-dimensional input resembling image-shaped data. Both, transformers and 2D CNNs can deal with multi-channel inputs such as multiple time-series data and multiple 2D images (i.e. RGB images), respectively. Thus, EEG data was reshaped to match the input layer of each model accordingly. We use the notion *d*_*n*_ to describe the dimensions of the reshaped datasets, when *n* is the dimension number. Below is an elaboration on the structure and size of the dataset making use of the parameters described in Table 1:

• Experiment 1: The input data matrix for the transformer model, *X*_*tr1*_, is 3-dimensional and of the size *d*_0_ × *d*_1_ × *d*_2_ = Tr  × (*N*_1_+*N*_2_) × *Channels* = Tr  × (*N*_1_+*N*_2_) × *E* = 3,753 × (150 + 171) × 60. Thus, a single trial passing through the model has size of *d*_1_ = *N*_1_+*N*_2_ (sequence length) and *d*_2_ = *E* (number of electrodes). For the 2D CNN model on the other hand, the data is shaped differently. The input data matrix for the 2D CNN model, *X*_*cnn1*_, is 4-dimensional and of the size *d*_0_ × *d*_1_ × *d*_2_ × *d*_3_ = Tr  × (*N*_1_+*N*_2_) × *E* × *Channels* = 3,753 × (150 + 171) × 60 × 1. Thus, a single trial passing through the model has size of *d*_1_ = 321 (sequence length), *d*_2_ = 60 (number of electrodes), and *d*_3_ = 1 (number of channels).
• Experiment 2: The input data matrix for the transformer model, *X*_*tr2*_, is 3-dimensional and of the size *d*_0_ × *d*_1_ × *d*_2_ = Tr  × (*N*_1_+*N*_2_) × *Channels* = Tr  × (*N*_1_+*N*_2_) × *E* = 5,418 × (150 + 171) × 60. Thus, a single trial passing through the model has size of *d*_1_ = *N*_1_+*N*_2_ (sequence length) and *d*_2_ = *E* (number of electrodes). For the 2D CNN model in the other hand, the data is shaped differently. The input data matrix for the 2D CNN model, *X*_*cnn2*_, is 4-dimensional and of the size *d*_0_ × *d*_1_ × *d*_2_ × *d*_3_ = Tr  × (*N*_1_+*N*_2_) × *E* × *Channels* = 5,418 × (150 + 171) × 60 × 1. Thus, a single trial passing through the model has size of *d*_1_ = 321 (sequence length), *d*_2_ = 60 (number of electrodes), and *d*_3_ = 1 (number of channels).

Note that the number of channels in the transformer model correspond to the number of electrodes. However, since the whole EEG dataset can be shaped in a single 2D matrix where its dimensions are the total sequence length by the number of electrodes, a single 2D channel is sufficient for the 2D CNN model.

### 2.6 Deep learning model architecture

We aim to build two robust classifiers that shall work at the single trial (epoch) level: the first is a binary classifier for detecting the presence of haptic delay during a discrete haptic feedback stimulation while the second is a multi-class classifier for detecting the level of haptic delay during a continuous haptic feedback stimulation. Herein, we explore using two different deep learning models, namely, 2D CNN and transformer. For both classifiers, we develop the same model with a single difference that lies in the size of the last dense layer which is essentially equal to the number of classes.

#### 2.6.1 2D CNN

The architecture of the 2D CNN model followed the DeepConvNet architecture proposed to handle EEG data classification (Schirrmeister et al., 2017). The architecture and design choices of the DeepConvNet model were particularly designed to suite specific characteristic of EEG data compared to image data. For example, EEG data are non-stationary time-series obtained through electrodes placed on the scalp which are fundamentally different than natural images commonly used to train 2D CNNs. Additionally, EEG data have low signal-to-noise ratio which makes the task-related features hard to capture. The DeepConvNet consist of four blocks where each block has a series of convolutional, batch normalization, max pooling and dropout layers. The first block is an exception in which two convolotional layers are used consecutively to extract temporal and spatial features, respectively. All non-linear activation functions in the network are based on exponential linear units (ELU) (Clevert et al., 2015) which was found to heavily impact the performance of the network compared to Rectified Linear activation Unit (ReLU). ELU activation function differs from ReLU in its response to negative inputs and is described by the below equation:

$$f\left(x\right)=\left\{\begin{array}{cc} x\; & \text{for x }>\text{ 0}\\ e^{x}-1\; & \text{for x }\leq \text{ 0} \end{array}\right.$$

Since the sampling frequency in this work differs from that in the DeepConvNet paper (125 vs. 250 Hz), some of the model parameters were altered such as the kernel and the pooling sizes.

#### 2.6.2 Transformer

Transformer is a model that was originally proposed in the natural language processing (NLP) domain for text data processing (Vaswani et al., 2017). Soon enough, the transformer model was adapted to deal with other types of data such as time-series data (Li S. et al., 2019) as well as images (Dosovitskiy et al., 2020). The strength of transformers lies mainly in the attention mechanism it employs in learning important features from the raw data. Attention mechanism operates in such a way that the model is able to focus on different parts of the input concurrently and highlight relationships between them in an attempts to capture higher order dependencies (Zhao et al., 2021). The model shall be able to attend to features from the raw EEG data as well as from the wavelet transforms concurrently. This capability of transformers is called self-attention and is calculated through the below equation:

$$Attention\left(Q,K,V\right)=softmax\left(\frac{QK^{T}}{\sqrt{d_{k}}}\right)V$$

where *Q*, *K* and *V* are the query, key and value matrices and *d*_*k*_ is the length of a single key vector (Vaswani et al., 2017).

In this work, we use a transformer model that consists of four consecutive encoder blocks, followed by a global average pooling layer and two dense layers. For each encoder block, the head size, also known as the key dimension was set to 256, the number of heads was set to four and the dropout rate for the attention layer is set to 0.25. The feedforward part of the encoder consisted of a dense layer of size 128 and a dropout rate of 0.4. For all activation functions in the dense layers, ELU function was used. The choices of these parameters are based on the outcome of an optimization process by Optuna framework (Akiba et al., 2019) as will be described in *Transformer*.

#### 2.6.3 Ensemble model

**FIGURE 4:**
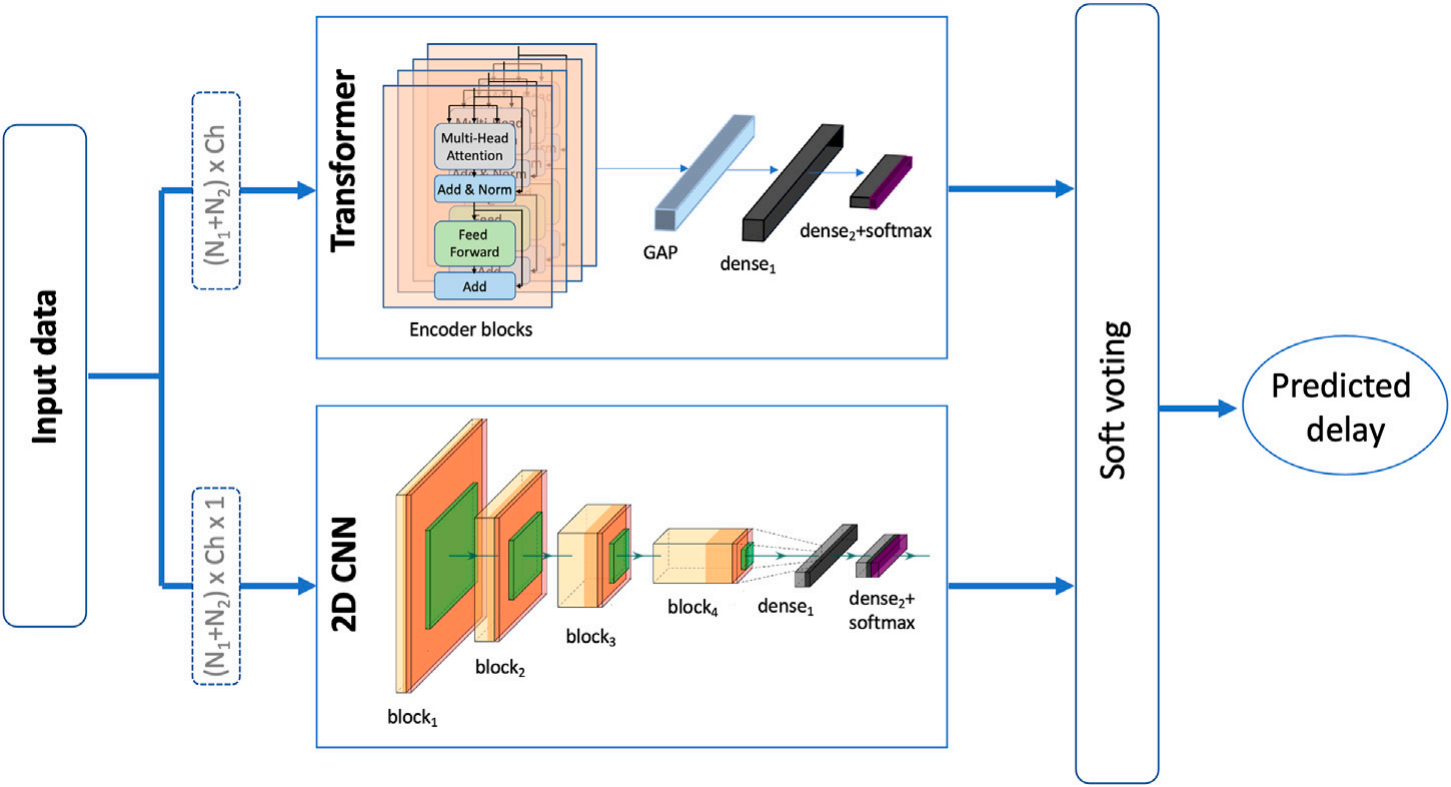
An illustration of the proposed ensemble model that takes a soft vote of a 2D CNN model (Deep ConvNet) and a transformer model to predict the presence/level of haptic delay.

An ensemble model consisting of both, the 2D CNN and the transformer models is also examined. The output of both models is combined in a soft voting manner such the probabilities of each class are averaged and the predicted delay is based on the class with the highest probability. Soft voting considers each voter’s degree of certainty rather than just their binary input. The proposed ensemble model is depicted in Figure 4. This model was examined and tested for both experiments with the only difference being in the size of the final dense layer of the transformer as well as the 2D CNN model.

### 2.7 Hyperparameter optimization and training

To optimize the training hyperparameters as well as the transformer model parameters, we used Optuna framework particularly designed to optimize the choices of hyperparameters for machine learning. We define a reasonable search spaces for each of the parameters we intend to optimize from which a sampling algorithm will be used to pick different combination of parameters at every run. Sampling algorithms are superior to random grid search such that the sampler tries to pick a combination of parameters that are likely to increase the accuracy based on the outcome of the previous runs. In this work, we used the Tree-structured Parzen Estimator (TPE) algorithm for sampling parameters from the search spaces. TPE is the default sampling method and generally performs well for runs less than 1,000. We performed 200 runs to search for the best parameters for the transformer model previously mentioned in *Hyperparameter optimization and training*. As for the training hyperparameters, another 200 runs were conducted to optimize for: batch size, learning rate and the optimizer type. The below hyperparameters were found to perform best for each of the models:

• 2D CNN: Learning rate is 1e-4, optimizer is ADAM, and batch size is 64.
• Transformer: Learning rate is 1e-3, optimizer is ADAM, and batch size is 64.

Under-fitting was addressed by increasing the complexity of the models through increasing the number of convolutional layers and the number of encoder blocks in the 2D CNN and transformers respectively, up to a point beyond which the increase did not help improve the validation accuracy. On the other hand, over-fitting was addressed by introducing dropout layers at the end of every convolutional block and after the attention layer in the 2D CNN and the transformer model, respectively. We used binary and categorical cross entropy functions for loss calculation for experiment 1 and experiment 2, respectively. For the training process, we first shuffle the whole dataset from all subjects and we split it to 80 and 20% for training and testing the model, respectively. To optimize the model weights, we then use 5-fold cross-validation where one fold is used for validation and weights optimization in every run. We ran the training for 300 epochs and the model with the best validation accuracy was used. The reported performance across the manuscript refers to the average performance across the five folds on the previously reserved testing dataset.

## 3 Results

For both experiments, we conducted six primary tests to compare the performance of the three suggested models (2D CNN, transformer, and ensemble) when using just the raw EEG data vs. embedding the wavelet transform coefficient. The findings of these tests for both experiments are summarized in the following sections.

### 3.1 Experiment 1: Detecting the presence of delay during a discrete haptic feedback

**FIGURE 5:**
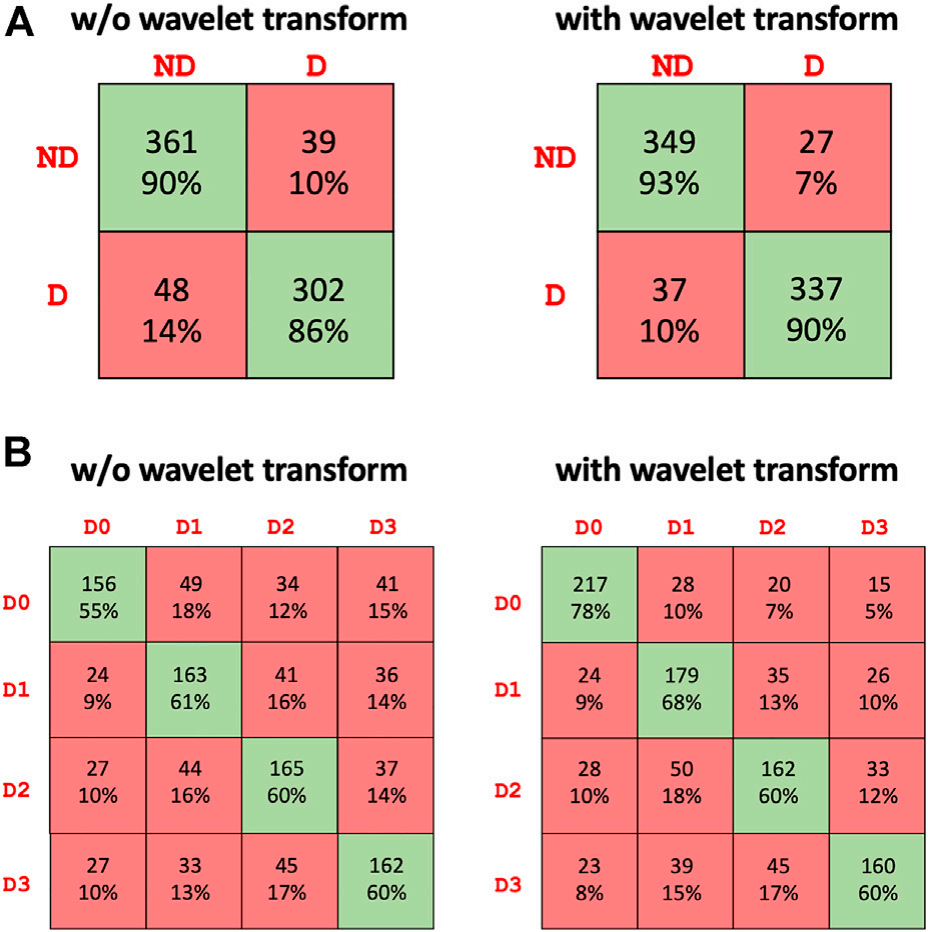
Confusion matrix of the best performing model (ensemble) for the **(A)** presence of haptic delay detection [two classes: No delay (ND) and Delay **(D)**] and **(B)** level of haptic delay classification [four classes: D0 (No delay), D1 (mild delay), D2 (moderate delay), D3 (severe delay)].

Table 2 summarizes the results of the conducted tests for detecting the presence of haptic delay regardless of the type of haptic interaction (passive vs. active). It can be observed that including the wavelet coefficients in the dataset improves the performance of all three models. Particularly, the performance of the transformer model is noticeably higher when wavelet coefficients are employed. All the models were trained and cross validated using the same settings. The highest accuracy achieved is 0.9142 by the ensemble model when wavelet coefficients are added to the dataset. The confusion matrix of the ensemble model is shown in Figure 5A. The confusion matrix shows that including the wavelets coefficients increases the true positive and true negative rates and reduces the false positives and false negative rates. Both, the precision and recall are boosted fas can be seen from Table 2.

**TABLE 2:** Performance of the two models and their ensemble on the presence of haptic delay dataset (two classes).The reported figures are the mean and standard deviation for 5-fold cross validation obtained on the test set.

Model	Accuracy		Precision		F1 score	
	w/o wavelet	with wavelet	w/o wavelet	with wavelet	w/o wavelet	with wavelet
2D CNN	0.8822 ± 0.0112	0.8940 ± 0.0076	0.8800	0.8950	0.8800	0.8950
Transformer	0.8348 ± 0.0069	0.9102 ± 0.0163	0.8300	0.9100	0.8300	0.9100
Ensemble	0.8897 ± 0.0106	**0.9142** ± 0.0157	0.8800	0.9150	0.8800	0.9150

### 3.2 Experiment 2: Classification of the level of haptic delay during a continuous haptic feedback

Table 3 summarizes the results of the conducted tests for detecting the level of the haptic delay during a continuous haptic feedback stimulation. This problem is obviously harder as the number of classes is four as opposed to only two for the delay presence detection. The same observation is valid regarding the positive impact of the wavelet transforms on the performance of all models, specifically the transformer model. The ensemble model outperforms the standalone models with an average accuracy of 0.6625 across the five folds. The confusion matrix of the ensemble model is shown in Figure 5B. The ability to accurately categorize all haptic delay levels has improved, with the no delay level (*D*_0_) showing the highest increase in detection accuracy.

**TABLE 3:** Performance of the two models and their ensemble on the levels of haptic delay dataset (four classes).The reported figures are the mean and standard deviation for 5-fold cross validation obtained on the test set.

Model	Accuracy		Precision		F1 score	
	w/o wavelet	with wavelet	w/o wavelet	with wavelet	w/o wavelet	with wavelet
2D CNN	0.5856 ± 0.0112	0.6339 ± 0.0097	0.5975	0.6320	0.5975	0.6250
Transformer	0.5223 ± 0.0105	0.6118 ± 0.0046	0.5325	0.6075	0.5325	0.6100
Ensemble	0.5959 ± 0.0044	**0.6625** ± 0.0067	0.6125	0.6625	0.6125	0.6625

## 4 Discussion

This work proposed a novel ensemble approach for modeling the presence and level of perceived haptic delay during a discrete and a continuous force feedback, respectively. The ensemble of 2D CNN and transformer models was able to achieve a mean accuracy of 0.9142 and 0.6625 in successfully detecting the presence and the level of haptic delay, respectively, on a single trial basis. The results of the study suggest that the EEG data contains rich information about the haptic delay experience even at the single trial level. The achieved detection accuracy is way beyond the chance level of 50 and 25% for two class and four class classification problems, respectively.

The detection accuracy of haptic delay in a four-level setting is clearly significantly lower than that of a binary setting. Identifying the level of haptic delay is a more challenging problem due to several reasons: 1) It is a multiclass problem where more intricate representations are to be learnt to distinguish between the four delay levels 2) We are not attempting to classify four different cognitive functions. Instead, we are trying to distinguish between four intensities of the same cognitive function (haptic delay perception) in which there is a resemblance between the adjacent delay levels. This is evident from the confusion matrix in Figure 5B where the false negatives and positives are generally higher closer to the target class. 3) Detecting a haptic delay during a continuous force feedback stimulation could be less perceivable compared to experiencing delay during a discrete feedback.

Another important challenge in detecting the perception of haptic delay in general, regardless of the type of experienced haptic feedback, is that its perception is a higher cognitive function that occurs in a temporally localized time-frame, normally within few tens or hundreds of milliseconds (Alsuradi et al., 2022). Other classification problems such as emotion or texture classification have trial lengths that last for few seconds and could last for up to few minutes (Eldeeb et al., 2019; Zeng et al., 2019; Babushkin et al., 2021). Cognitive functions that last for longer period of time are more easily detected as the neural signature is spread across the trial duration. Additionally, robust features such as total power of different frequency bands (delta, theta, alpha, beta, gamma) across the trial length can be extracted using Welch (Welch, 1967) or Bartlett’s methods (Bartlett, 1948) which are quite effective towards the downstream classification task. Other examples from the literature on EEG-based four class classifiers that aim to classify higher cognitive functions show comparative performance. For example, several studies on emotion recognition (four emotional states) reported classification accuracy of 67% (Liang et al., 2019) 66% (Huang et al., 2017) and 62% (Li P. et al., 2019).

An interesting observation was the noteworthy increase in the detection accuracy when wavelet transform coefficients were taken into consideration and concatenated to the raw EEG data. Particularly, the false negatives of *D*_0_ sharply decreased as can be observed from Figure 5B. This result imply that wavelet transforms were able to highlight and extract features of synchronous stimulation (*D*_0_) which were otherwise not as detectable from the raw EEG data. The effectiveness of wavelet transform mainly lies in its ability to localize and extract features at various temporal and frequency locations (Rao, 1999; Samant and Adeli, 2000). Where other techniques in signal processing fall short or are ineffective, the wavelet transform is particularly good at expressing different features of signals, such as trends, discontinuities, and repetitive patterns which makes them very powerful for extracting features from non-stationary signals such as EEG data. This implies an accurate extraction of transient EEG features (Adeli et al., 2003) which are particularly present during the perception of haptic delay. Our previous studies (Alsuradi et al., 2021, 2022) confirm the presence of statistically significant differences across the delay conditions, which the current study corroborate by the above-the-chance levels of classification accuracy.

Assuredly, concatenating wavelet based features improved the detection accuracy with a large margin. However, one limitation of our approach is the lack of a systematic examination of the selected wavelets and their role in extracting particular neural features. We relied on an empirical approach for selecting the base-wavelets, however, a more systematic approach could yield a better performance and improve the model’s explainability. Another point is that our proposed model is trained based on inter-subject classification as opposed to intra-subject classification models (Müller-Gerking et al., 1999; Wang et al., 2012; Bae and Luck, 2018; Eldeeb et al., 2019) which are trained, tested and used on the data of a single subject only. Generally, inter-subject models must contend with greater data variability which introduces challenges related to learning subject-resilient features. Inter-subject models almost always perform worse than intra-subject models (Hajinoroozi et al., 2017) due to the challenges of learning subject-independent features. However, in inter-subject models, the validation procedure can have an impact on the learning curve of the model. One method is to use k-fold cross-validation on the combined data from all the subjects which we used in this study. Another way is to use leave-N-subjects-out method which splits the data on the basis of subjects. Both methods are used and reported in the literature (Roy et al., 2019; Joucla et al., 2021). The latter method usually generalizes better and thus is the second limitation of the study. Furthermore, given that most participants were recruited from one age group (18–25 years old), the results are not generalizable to other age groups (given how haptic perception varies across age groups). However, participants were drawn from a highly diverse racial backgrounds which makes the results generalizable from the racial background perspective.

## 5 Conclusion

This paper presents an ensemble deep-learning based model for the detection and severity level classification of haptic delay during discrete and continuous haptic feedback from single trial EEG data. The ensemble model comprises 2D CNN and transformer models. Raw EEG data and several of their wavelet transforms were used without crafting or manually extracting features; instead, the model relies on the self-attention mechanism and the automatic detection of features through CNN filters. The ensemble model showed a promising performance with an accuracy of 0.9142 ± 0.0157 and 0.6625 ± 0.0067 for the binary and multi-class classification problems, respectively.

For future work, we believe that the model that detects the level of haptic delay could further improve by incorporating other data modalities which could be relevant to the detection of the level haptic delay. For instance, force feedback data delivered by the haptic device or EMG data detected at the surface skin of the involved hand. Since the ensemble-based model is currently just dependent on the EEG data, adding other sensory modalities that are pertinent to haptic delay will greatly increase the detection accuracy. To get sufficient data for model’s training, this could require recruiting a considerably higher number of individuals. Lastly, it is possible to experiment with other features commonly used for physiological time-series data such as entropy related (Richman and Moorman, 2000; Wu et al., 2013) and dynamic features (Seto et al., 2015; Yuan et al., 2019).

## Data Availability

The raw data supporting the conclusion of this article will be made available by the authors, without undue reservation.
